# Thunder and Lightning

**Published:** 1916-10

**Authors:** 


### Thunder and Lightning.

In the registration area, lightning kills about 1 person per 200,000 population, annually or causes approximately one in every 3000 deaths. Most human beings and many lower animals are afraid of thunder and lightning, the fear being disproportionate to the actual danger. It appears, indeed, that we are dealing with a phobia rather than an actual fear in most cases. It is scarcely necessary to state that the actual danger is from lightning and not from the accompanying thunder yet, so far as we can judge, it is the latter which mainly produces the phobia, whether in human beings or animals. This is especially true of intelligent persons who realize the very slight proportionate danger from lightning and know, as a matter of scientific training that all danger is past, from the particular discharge of electricity, by an appreciable interval when they hear the thunder. Brontophobia is, therefore, a strictly correct expression. Numerous cases of marked hysteria occur during thunder storms and, so far as may be judged from the infrequent opportunities of comparison, the cases are much fewer from witnessing heat lightning or the rare instances in which "balls of fire" enter buildings without detonations.

If we recall the general rule that phobias originate early in human history or even antedate the arrival of man on the globe. it is not so strange that thunder rather than lightning should cause the fear. Visual sensations are almost constantly received except during sleep and those of unusual degree are less uncommon than analogous stimulation of the other special senses. Moreover, a sudden loud noise, usually does mean danger, at least potentially.

